# Review of *The Biogeography of Host-Parasite Interactions *by Serge Morand and Boris R Krasnov

**DOI:** 10.1186/1756-3305-4-34

**Published:** 2011-03-14

**Authors:** Jean-François Guégan

**Affiliations:** 1 Institut de Recherche pour le Développement, Montpellier, France

## Abstract

**Morand, S and Krasnov, B.R**. *The Biogeography of Host-Parasite Interactions*. Oxford University Press; 2010. 277 pages, ISBN 978-0-19-956134-6 (Hbk.), 978-0-19-956135-3 (Pbk.).

## Review

When starting to read the book, I first decided to come back to the definition of what is biogeography? Biogeography is the study of the distribution of living organisms spatially and temporally. The scope of this multi-disciplined field aims to reveal where organisms live, at what abundance, and why they are, or are not, found in a certain geographical area. The patterns of species distribution across geographical areas can usually be explained through a combination of historical factors such as speciation, extinction, continental drift and glaciation, in combination with the geographical constraints of landmass areas and isolation, and the available ecosystem energy supplies. Biogeography is also important in extrapolating the ripple effects of natural and man-made impacts on organism range and distribution. More generally, biogeography is the science that attempts to document and understand patterns of biodiversity, which means that biogeographers seek to understand the interactions between populations, species and ecological communities with their environment, in space and time.

Astonishingly, consideration of population and species health and viability is still rare in biogeography whereas the role of parasites in regulating host population abundance and in exterminating local species - thus what can make one species common and even abundant, and what can make another species rare - has received much attention over the past decade. The biogeography of some hosts may be influenced by the distribution of parasites, quite apart from the abiotic influences and, in contrast, the biogeography of parasites is determined by host suitability and availability and therefore by the geographical distribution of their host species. As already suggested [1], host-parasite systems may be viewed as keystones in biogeography, and this book by Serge Morand and Boris Krasnov is addressing this important, but largely neglected, issue in biogeography.

The book comprises five sections: (*i*) Historical Biogeography, (*ii*) Ecological Biogeography and Macroecology, (*iii*) Geography of Interactive Populations, (*iv*) Invasion, Insularity, and Interactions, and (*v*) Applied Biogeography. Instead of describing each one of these and the many (18) chapters constituting the book, I will concentrate this review on what I really liked, and disliked, in it. First, the two scientific editors succeeded in gathering together a very impressive list of authors (38 including the foreword, the introduction and conclusion), some, if not all, being among the leading scientists in the fields of host-parasite systems, ecology or biogeography. This resulted in a vast list of examples and case-studies ranging from obligate mutualists of plants to animal parasites and human pathogens. The diversity of studies we can read in this book is undoubtedly one of its major strengths. Graduate students, research scientists and lecturers, as well as a broader audience within the fields of geography, epidemiology and veterinary medicine, will find here a goldmine in which to dig for minerals to illustrate their lectures or to influence their own research. I have already used some of these studies to illustrate some of my lectures and conference presentations, and I can imagine that many readers will do the same. My main negative criticism, as recurrently found with this type of multi-authored book, concerns its inherent diversity of writing style, of viewpoints and opinions on what biogeography really is, and of the main objectives to be addressed for this type of published work among the different chapters. In addition, here and there, some wording and typographical errors still persist, suggesting that its production may have been done too rapidly.

A title for this book review could have been *Host-parasite biogeography, or simply patterns of sampling effort only? *Reading the book, and after having finished reading Chapter 10 "Gap analysis and the geographical distribution of parasites" by Mariah E. Hopkins and Charlie L. Nunn, I then decided to stop my review work for a while. Not because of too much work to do in other areas, but because this chapter reaches an important conclusion that patterns of sampling efforts might (strongly) impact our knowledge of host-parasite biogeography. Since curiously a pdf file of this book can be easily downloaded from the Web, I checked how many times the sampling effort, sampling artefact or sampling effect expressions were used in the previous chapters and even in those subsequent. The term sampling(s) is used on very rare occasions but with another explanation, and it is used on page 90 only to discuss its confounding, perverse effect on parasite data. From chapter 11 to the conclusion, the term sampling appears 3 times but again for another reason, and in the Conclusion on page 268, by the editors, to re-discuss the importance of sampling in the biogeography of host-parasite interactions.

This chapter by Hopkins and Nunn is at the heart of what is, or what should be, the biogeography of host-parasite interactions, i.e., greatly influenced by geographically inconsistent sampling patterns (in terms of unexplored regions, unsampled host species, heterogeneous sampling efforts,...). This chapter in itself is questioning the validity of a biogeographical perspective in studies of host-parasite biogeography, when not checking for perverse effects exerted by sampling biases in space. Interestingly, it also suggests that gap analysis can be applied to guide spatial patterns in host-parasite systems sampling efforts. The above comments must be seen in the light of the next Chapter 11 "In the hosts' footsteps? Ecological niche modelling (ENM) and its utility in predicting parasite distributions" by Eric Waltari and Susan L. Perkins. Using ENM, the authors explore the distributions of parasites and their hosts, based on the attributes of some chosen bioclimatic, environmental and biological parameters. Several illustrations are given, and notably that of a tritrophic model, i.e., a tortoise, a tick and an apicomplexan pathogen; an invasive host species, the ant *Solenopsis invicta*, and its two microsporidian species; and 10 *Anopheles *species and human malaria in Africa. To come back to my earlier comments, it seems, based on my own survey of the literature, that we are dramatically lacking such examples of the geographic distributions of one host species, its potential vector species and its pathogen species over their overall ranges of distribution. In my opinion, this is exactly the research direction that the biogeography of host-parasite interactions should take. One needs to select some host species or host taxa, and to explore over their entire geographical ranges the distribution of their associated organisms like vectors, reservoirs and parasites. This will permit a cross-checking of all these with epidemiological data (prevalence, incidence, morbidity, mortality,...) and organism life-history traits (host susceptibility, nutritional status, pathogen virulence,...) in order to better understand host-disease interactions in a geographical dimension. Morand and Krasnov are proposing some interesting research perspectives that should be taken into account by the community within this field of research in the near future.

To end this review, the reader will find in this book some excellent chapters, which will certainly provide stimulation and creativity for new research perspectives in host-parasite biogeography. Essentially, because sampling bias is, as usual, the demon in ecology, evolution and biogeography, host-parasite biogeography will need first to exorcize it using optimization techniques on where to prioritize the sites to visit, the order in which to visit them, the host taxa and even the parasite taxa to sample in a time where we are describing more and more disease agents [2], and more specifically viruses and protozoans.

## Competing interests

The author declares that they have no competing interests.
